# Bio-derived NiO nanoparticles from *Colocasia esculenta* leaf extract with enhanced antibacterial activity and efficient photocatalytic degradation of methylene blue

**DOI:** 10.1039/d5ra08840b

**Published:** 2026-01-15

**Authors:** Tekileab Engida Gebremichael, Endale Tesfaye, Teshome Abebe, Alemnesh Bekele, Zerfu Haile Robi, Tamirat Engida Gebremikael

**Affiliations:** a Chemistry Department, Gambella University Gambella Ethiopia tekileab2009@gmail.com tekileab@gmu.edu.et; b Chemistry Department, Jimma University Oromia Ethiopia; c Chemistry Department, Oda Bultum University Ciro Ethiopia; d Mechanical Engineering Department, FDRE Technical and Vocational Training Institute (FTVTI) Addis Ababa Ethiopia

## Abstract

*Colocasia esculenta* leaf extract was utilized for the green synthesis of nickel oxide nanoparticles (NiO NPs) through an easy, phytochemical-mediated, environmentally friendly reduction method. Fourier transform infrared (FTIR) spectroscopic analysis of the nanoparticles revealed that the phytochemicals served as both capping and reducing agents, and the morphology of these nanoparticles was studied using scanning electron microscopy (SEM). The surface plasmon resonance (SPR) peak was observed at 348 nm, the optical band gap was 3.45 eV based on UV-visible analysis, and the crystallite size of the synthesized NPs was 26.89 nm, as evidenced by X-ray diffraction (XRD) analysis. Strong antimicrobial potential was demonstrated against *Staphylococcus aureus*, *Salmonella typhi* and *Candida albcans*, producing inhibition zones of 19.00 ± 0.94 mm, 17.33 ± 0.62 mm and 20.67 ± 0.58 mm, respectively. Additionally, under optimal conditions (30 mg catalyst, 10 mg L^−1^ dye, pH 8, 80 min), NiO NPs demonstrated effective photocatalytic degradation of methylene blue under natural sun irradiation, attaining up to 96% degradation. Interestingly, after three consecutive cycles, the nanoparticles retained 85% of their photocatalytic activity, demonstrating exceptional stability and reusability. These results demonstrate the promise of *Colocasia esculenta*-mediated NiO NPs as multipurpose, biocompatible, and reasonably priced nanomaterials for biomedical and environmental remediation applications.

## Abstract 2

Eco-friendly NiO nanoparticles from *Colocasia esculenta* leaf extract show enhanced antibacterial activity and efficient dye degradation, proving useful for biomedical and environmental tasks.

## Graphical abstract

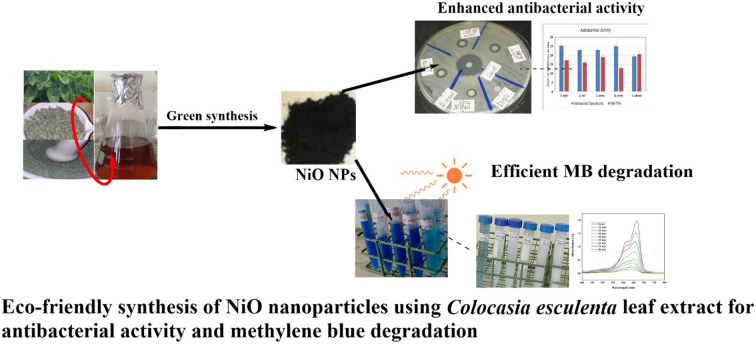

## 1 Introduction

Environmental pollution, especially water contamination, has become a major worldwide concern.^1^ The increasing discharge of synthetic dyes and microbial contaminants into water bodies poses significant environmental and health challenges.^2^ Organic colors from mining, textile, paper, food processing, cosmetics, pharmaceuticals, and leather industries are among the many pollutants that contaminate water.^3^ Because of its toxicity, carcinogenicity, potential for bioaccumulation, and resistance to biodegradation, methylene blue (MB), a common dye, is especially concerning.^4^ In order to remove colors from wastewater, a number of techniques have been used, such as adsorption, ion exchange, membrane filtering, electrochemical treatment, and photocatalysis.^5^ Because of its great efficiency, affordability, and environmental friendliness, photocatalytic degradation has become one of the promising methods.^6^ Metal-based nanoparticles can simultaneously break down organic molecules when exposed to light, and they have garnered attention for their exceptional photocatalytic activity.^7^ Meanwhile, microbial, bacterial, fungal, viral, and parasitic infections continue to pose serious health risks worldwide, especially in underdeveloped nations where these issues exacerbate due to antibiotic resistance and lack of available medications.^8,9^ The management of infectious diseases has improved since the discovery of antimicrobial drugs,^10,11^ but the emergence of resistance necessitates the use of alternative antimicrobial techniques.^12^

Nanotechnology has emerged as an effective solution for these biomedical and environmental threats.^13,14^ Nanoparticles exhibit remarkable physical and chemical properties due to their small size and high surface area.^15^ Among the different metal-based nanostructures,^16,17^ nickel oxide (NiO) nanoparticles have attracted considerable attention due to their unique physicochemical properties, making them effective photocatalysts for dye degradation and promising antibacterial agents.^6,18,19^

Recently, plant extract-based green synthesis techniques have become environmentally benign and sustainable substitutes to conventional chemical methods, offering advantages such as reduced toxicity and cost-effectiveness and providing natural capping and stabilizing agents.^20,21^ Because the phytochemicals in plant extracts can function as reducing agents, they are promising biological templates in this regard.^2^ However, there are no reports on the antibacterial and photocatalytic capabilities of NiO nanoparticles made from *Colocasia esculenta* extract.^22^

*Colocasia esculenta*, commonly known as Godere (*in Amharic*), is a kind of root vegetable that is inexpensive and widely available. The plant leaves are notable for their significant therapeutic and nutritional value. They contain a wealth of substances with potential medical uses, including terpenoids, alkaloids, tannins, flavonoids, and saponins.^23^ These biomolecules possess strong reducing and capping ability, suggesting that the extract of *Colocasia esculenta* leaves can act as an excellent bioreductant for controlling the growth and morphology of NiO NPs.^24^ However, reports on the synthesis of NiO NPs for simultaneous antibacterial activity and photocatalytic degradation of MB dye under visible-light irradiation are extremely limited. To the best of our understanding, no prior research has documented the environmentally friendly production of NiO NPs using *Colocasia esculenta* leaf extract, and the combined testing of their photocatalytic degradation of dyes and antibacterial activity has not been investigated. Considering this, the present research conducts an environmentally friendly synthesis of NiO NPs, employing the leaf extract from *Colocasia esculenta*, and evaluates their antibacterial activity and photocatalytic degradation efficiency against methylene blue dye.

The phytochemical composition of the extract and the structural properties of NiO NPs were examined using FT-IR, UV-vis spectroscopy, SEM, and XRD analysis. The antimicrobial performance was tested against *B. cereus*, *S. typhi*, *E. coli*, *C. albicans*, and *S. aureus*, while the photocatalytic degradation of MB was monitored under visible-light irradiation through UV-vis spectroscopy. This study demonstrates that synthesized NiO NPs can serve as a dual-functional nanomaterial for disinfection and wastewater treatment, underscoring their potential for use in biomedical and environmental applications.

## 2 Experimental section

### 2.1 Chemicals and apparatus

Nickel nitrate hexahydrate (Ni(NO_3_)_2_·6H_2_O, ≥99.9%), potassium permanganate (KMnO_4_), methylene blue (MB), nutrient agar, sodium hydroxide, sulfuric acid, nitric acid, chloroform, bismuth nitrate (Bi(NO_3_)_3_), potassium iodide, glacial acetic acid, ferric chloride, and ammonia solution were used as received. Fresh *Colocasia esculenta* leaves (*Godere in Amharic*) were collected near Gambella University.

Apparatus included conical flasks, Petri dishes, digital balance, magnetic stirrer, mortar and pestle, oven, hot plate, centrifuge, glass rods, droppers, graduated cylinders, Erlenmeyer flasks, cuvettes, refrigerator, Teflon-lined autoclave, hot air drier, and furnace (D550, Ney Vulcan, USA).

### 2.2 Preparation of *Colocasia esculenta* leaves

Pure water was used to completely wash the fresh leaves of *Colocasia esculenta*, followed by distilled water, and allowed to air dry at room temperature. Using a pestle and mortar, 25 g of leaves were ground. The extract was prepared as follows: 10 g of chopped leaves were boiled in 100 mL of water at 60 °C for 20 min under continuous stirring. After filtering the mixture through Whatman no. 1 filter paper, the clear extract was kept at 4 °C to be employed as a reducing agent for the manufacture of NiO nanoparticles.^25,26^

#### 2.2.1 Preliminary phytochemical screening

Phytochemical groups such as alkaloids, flavonoids, phenols, terpenoids, tannins, saponins, and glycosides were tested according to standard methods.^27,28^

##### 2.2.1.1 Alkaloids

1 mL of *Colocasia esculenta* leaf extract was mixed with 1 mL of 1.5% HCl, followed by 1 mL of Wagner's reagent. The appearance of a yellow or brown precipitate indicated the presence of alkaloids.

##### 2.2.1.2 Saponins

2 mL of *Colocasia esculenta* leaf extract was mixed with 10 mL distilled water, and then shaken for 15 min. Foam formation (1 cm) indicated the presence of saponins.

##### 2.2.1.3 Flavonoids

1 mL extract was treated with 5 drops of NaOH. The appearance of an intense yellow color confirmed the presence of flavonoids.

##### 2.2.1.4 Tannins and phenolics

1 mL extract was treated with 5% neutral ferric chloride. The appearance of a bluish-black or dark blue color indicated their presence.

### 2.3 Synthesis of NiO nanoparticles

NiO NPs were synthesized *via* a green approach using nickel nitrate hexahydrate. A 0.1 M nickel nitrate solution was prepared by dissolving 3 g of Ni(NO_3_)_2_·6H_2_O in 100 mL of distilled water. Subsequently, 30 mL of *Colocasia esculenta* leaf extract was added to 100 mL of the nickel nitrate solution and stirred for 5 min, giving an initial pH of 3.2. To determine the optimal precipitation condition, the reaction pH was screened from acidic to basic ranges (pH 4–11). These preliminary tests showed that pH 9 produced the most uniform and stable precursor, resulting in well-defined NiO nanoparticles after calcination. Therefore, the pH of the reaction mixture was carefully adjusted to 9 by the dropwise addition of 2 M NaOH while stirring continuously. A concentrated NaOH solution was used to achieve precise pH control with minimal dilution of the reaction mixture. Once the pH reached 9, the mixture was stirred for 1 h at 60 °C until a greenish slurry was formed. The precipitate was then collected by centrifugation at 4000 rpm for 30 min, washed three times with distilled water and ethanol, and dried at 80 °C. Finally, the dry precursor was calcined at 500 °C for 2 h to yield crystalline NiO nanoparticles.^6,29,30^

### 2.4 Optimization of the synthesis parameters

To guide the optimization of the NiO nanoparticle synthesis, the key findings by Kannan *et al.* (2020) are summarized in Table 1. Kannan *et al.* demonstrated that the synthesis parameters, including the plant extract volume, precursor concentration, pH, temperature, and reaction time, strongly influence nanoparticle properties such as the particle size, dispersity, crystallinity, and UV-vis absorption characteristics.^25^ One parameter was varied at a time while keeping others constant to achieve nanoparticles with sharp peaks, small particle size, and narrow wavelength in UV-vis spectra. Optimized nanoparticles were stored in labeled containers for further characterization, antimicrobial testing, and dye degradation experiments.

**Table 1:** Summary of the photocatalytic and biomedical activities of nanostructured metal oxides (from ref. 25) upon nanoparticle optimization

Parameter investigated	Variation studied	Observed effects on nanoparticles (ref. 25)	Relevance to the current study
Precursor concentration	Low → high	Higher concentration increased the particle size and broadened the UV-vis absorption peaks due to the faster nucleation and agglomeration	Helped determine the suitable Ni(NO_3_)_2_ concentration to maintain the small size and sharp peaks
Plant extract volume	Small → large	Increased extract volume provided more phytochemicals, improving the reduction and capping, leading to smaller and more stable NPs	Guided optimization of the extract amount (30 mL) for controlled NiO NPs formation
pH of the reaction mixture	Acidic → basic	Higher pH accelerated hydrolysis and nucleation; pH ∼9–10 produced smaller, uniform, well-dispersed NPs	Supports the selection of pH 9 as optimum for NiO NPs synthesis
Temperature	Room temp → elevated	Higher temperature increased the crystallinity, but excessive heating led to particle growth and aggregation	Informed selection of 60 °C synthesis temperature before calcination
Reaction time	Short → extended	Longer reaction improved the formation and stabilization, but a very long reaction time caused secondary growth	Helped define the 1 hour reaction time for controlled particle size and stability
Optical properties (UV-vis)	Varies with parameters	Sharp, narrow peaks indicate monodispersity; broad peaks reflect agglomeration or polydispersity	Used as criteria to judge the optimized NiO NPs in this study
Final recommendation	—	Optimal conditions produce nanoparticles with small size, narrow wavelength range, and good stability	Used as a benchmark for selecting the optimized synthesis conditions for NiO NPs

### 2.5 Characterizations

#### 2.5.1 UV-vis spectroscopy

The optical characteristics of the NiO nanoparticles were measured using a Shimadzu UV-2600 spectrophotometer in the wavelength range of 200–800 nm. A 1 mg mL^−1^ aqueous dispersion was sterilized with under ultrasonic treatment for 10 min before measuring in a 1 cm quartz cuvette.

#### 2.5.2 FTIR spectroscopy

Functional groups were investigated using a PerkinElmer Spectrum FTIR spectrometer in the 4000–400 cm^−1^ range, with 32 scans at 4 cm^−1^ resolution. 200 mg of KBr and 2 mg of sample were mixed together for KBr pellet readings.

#### 2.5.3 XRD

A Bruker D8 Advanced diffractometer (Cu Kα, *λ* = 1.5406 Å) was used to determine the crystallinity at 40 kV, 30 mA, in the 2*θ* range of 10–80° with a step size of 0.02°. The powdered samples were similarly packed on a flat holder. The Debye–Scherrer formula is used to compute the crystallite size (*D*) (eqn (1)).1
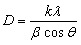
where *k* ≈ 0.9, *β* is the FWHM in radians, *λ* is the X-ray wavelength, and *θ* is the Bragg angle.

#### 2.5.4 SEM

The morphology was investigated using a JEOL JSM-6510 SEM at 15 kV. After drying, the nanoparticles were coated with gold and placed onto carbon tape. Images were acquired at 600–4400× magnification and the surface morphology was analyzed *via* a compositional software.

#### 2.5.5 Zeta potential analyzer

Zeta potential measurements were carried out using a Malvern Zetasizer Nano ZS (Malvern Instruments, UK) at room temperature. The point of zero charge (PZC) of the NiO nanoparticles was determined from zeta potential measurements as a function of pH. A suspension was prepared in distilled water, and the pH was adjusted from 2 to 12 using 0.1 M HCl or NaOH. Measurements were performed using a zeta potential analyzer in triplicate, and the PZC was identified as the pH at which the zeta potential crossed zero.

### 2.6 Antimicrobial activity

#### 2.6.1 Antibacterial activity

The agar disc diffusion method was performed on nutrient agar against Gram-negative and Gram-positive bacteria. Overnight bacterial cultures were adjusted to 1–2 × 10^8^ CFU mL^−1^. 6 mm discs soaked in NiO NP solutions were placed on plates inoculated with *B. cereus*, *E. coli*, *S. typhi*, and *S. aureus*. At 37 °C, plates were incubated for 24 h. The zone of inhibition (ZOI) was measured in millimeters and the experiments were performed in triplicate.^31–34^

#### 2.6.2 Antifungal activity

Fungal strain *C. albicans* was swabbed onto Muller–Hinton agar. Wells were created with a sterile cork borer and treated with NiO NP solutions, where DMSO served as the control. Plates were incubated at 27 °C for 24 h and the ZOI was measured.^35,36^

### 2.7 Photocatalytic activity

Photocatalytic degradation of methylene blue (MB) was conducted using 10 mg NiO NPs in 100 mL of 5 ppm MB solution under 250 W UV light irradiation. To ensure accurate mixing, the solution was continuously air-bubbled. The following formula (eqn (2)) was used to determine the percentage of dye degradation:^37–39^2
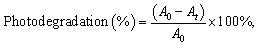
where *A*_0_ and *A*_t_ are the initial and final absorbances, respectively.^36,40^

## 3 Results and discussion

### 3.1 Phytochemical screening of *Colocasia esculenta* leaf extract

**Fig. 1:**
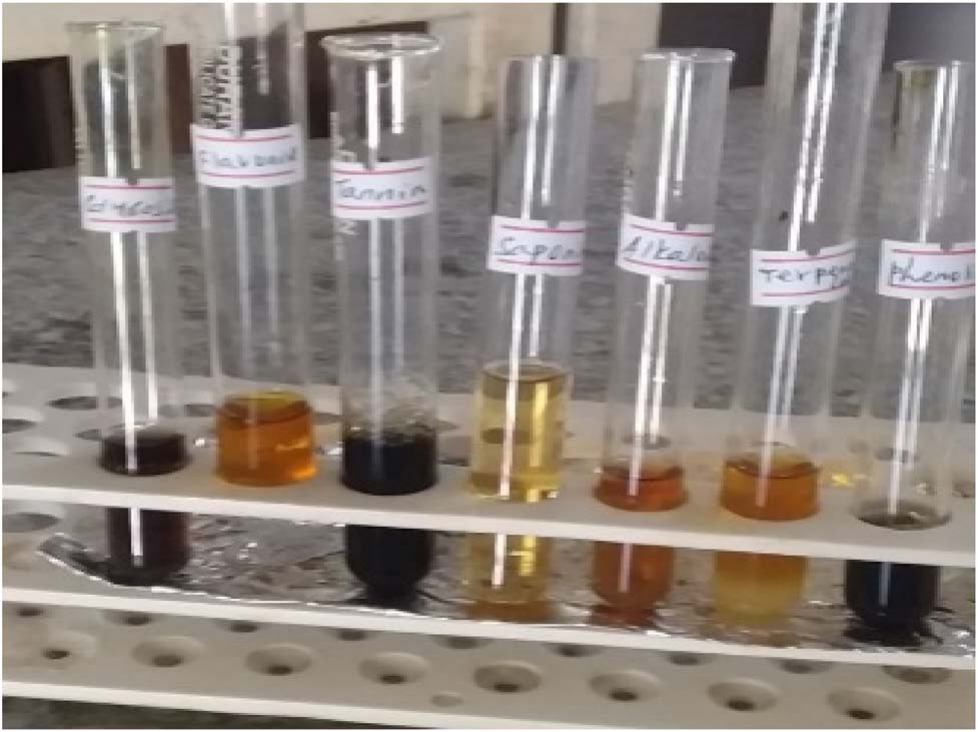
Phytochemical analysis of the *Colocasia esculenta* leaf extract.

Phytochemical screening was conducted to identify the bioactive compounds present in *Colocasia esculenta* leaf extract. These phytochemicals can serve as reducing agents during the synthesis of NiO NPs. The qualitative analysis (Fig. 1 and Table 2) indicates the presence of secondary metabolites, including polyphenols, flavonoids, saponins, tannins, alkaloids, glycosides, and terpenoids. The presence of phenolic compounds was confirmed by the appearance of a dark blue-black colour upon the addition of ferric chloride, indicating a significant concentration of these metabolites. The development of a yellow hue with NaOH confirmed the presence of flavonoids. The bio-reduction of Ni^2+^ ions depends on these components. The observed phytochemicals and their relative abundance are listed in Table 2.

**Table 2:** Phytochemical components of the *Colocasia esculenta* leaf extract^a^

Sl. no.	Phytochemicals	Observation in tube (color)	Inference	Classification
1	Flavonoids	Yellow-brown color	Clear positive	++
2	Alkaloids	Brown-orange coloration	Weak positive	+
3	Phenols	Dark coloration	Strong positive	+++
4	Saponins	Yellow solution with froth/foam	Positive	++
5	Tannin	Black/dark brown	Strong positive	+++
6	Glycoside	Brownish solution	Moderate positive	++
7	Terpenoids	Yellow-orange color	Weak positive	+

a “+” = low, “++” = moderate, “+++” = high, based on the color intensity observed in the standard assays compared to the reference controls.

The findings are in line with earlier research,^34^ suggesting that the leaf extract of *Colocasia esculenta* contains alkaloids, saponins, tannins, flavonoids, phenols, and glycosides that aid in the stabilization and reduction of NiO NPs. FT-IR spectral analysis further supported these conclusions.

### 3.2 UV-vis spectral analysis of NiO nanoparticles

**Fig. 2:**
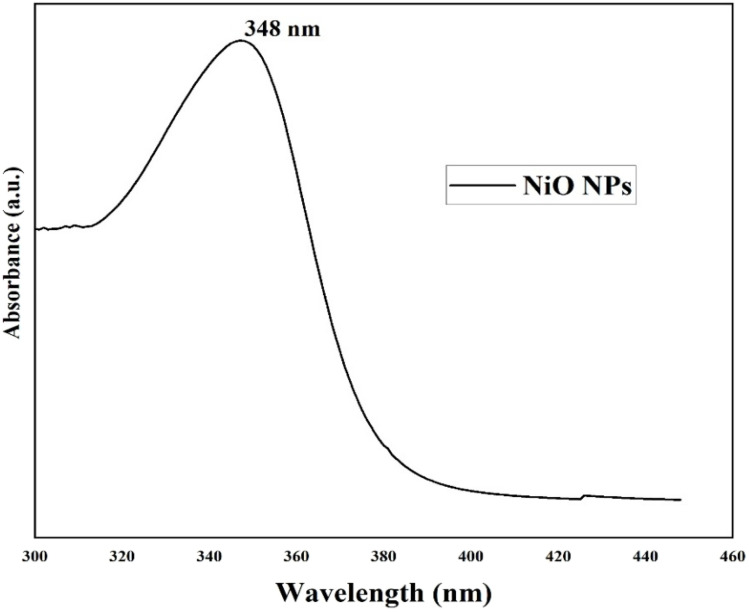
UV-vis absorption spectrum of NiO NPs showing the SPR peak at 348 nm.

**Fig. 3:**
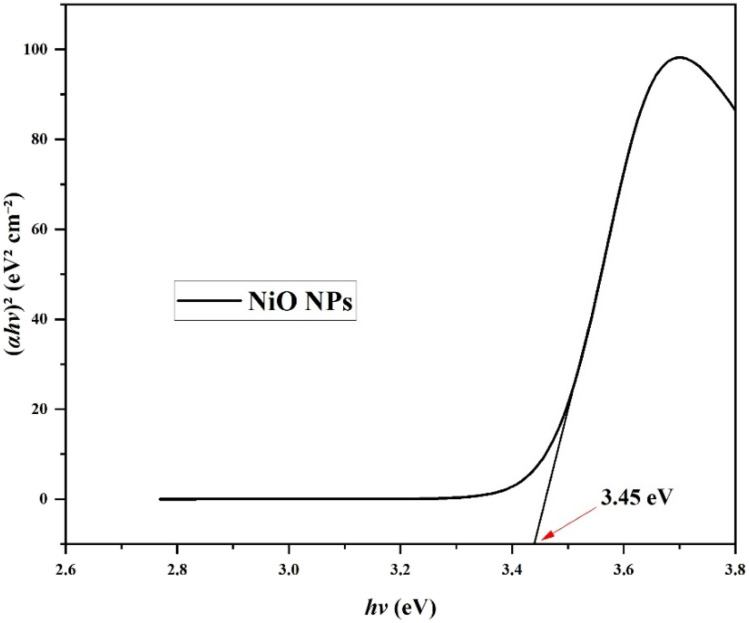
Tauc plot ((*αhν*)^2^*versus* photon energy (*hν*)) used to estimate the optical band gap of NiO NPs.

UV-vis spectroscopy, an effective tool for analyzing the synthesis and stability of NPs, was used to verify the formation of NiO nanoparticles. NiO NPs have a distinct surface plasmon resonance (SPR) peak at 348 nm, as seen in Fig. 2. This peak is blue-shifted in comparison to bulk NiO (*λ*_max_ = 362 nm). This is likely due to surface defects and capping effects from the *Colocasia esculenta* leaf extract, which can modify the electronic structure and influence the absorption edge.^41,42^ The collective oscillation of the nanoparticles' conduction electrons under incoming light is what causes the SPR. The measured absorption, which displays SPR bands in the 330–350 nm range, is in line with previously reported NiO NPs generated from a variety of bio-sources. The sharp and narrow nature of the SPR band suggests uniform particle size distribution and high stability of the synthesized NiO NPs. Tauc's approach was used to measure the optical band gap (*E*_g_) of NiO NPs (Fig. 3). The Tauc relation is expressed in eqn (3):

3 (*αhν*)^2^ = *A*(*hν* − *E*_g_),

where *A* is a constant, *α* is the optical absorption coefficient, *hν* is the photon energy, and *n* = 2 for the allowed direct transitions of the electron. The photon energy was calculated from the wavelength *λ* using eqn (4).4
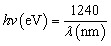


Extrapolation of the linear portion of the (*αhν*)^2^*versus hν* plot yielded a band gap. UV-vis analysis shown that NiO NPs synthesized here have a direct band gap of approximately 3.45 eV, which is somewhat greater than the 3.25 eV reported in earlier studies using *Senna auriculata*-mediated synthesis. The increased band gap may be attributed to the smaller particle size.^43^

The relatively large band gap contributes to enhanced surface charge and stability, which can improve the photocatalytic and antimicrobial performance of the NiO nanoparticles.

### 3.3 FTIR analysis of NiO nanoparticles

**Fig. 4:**
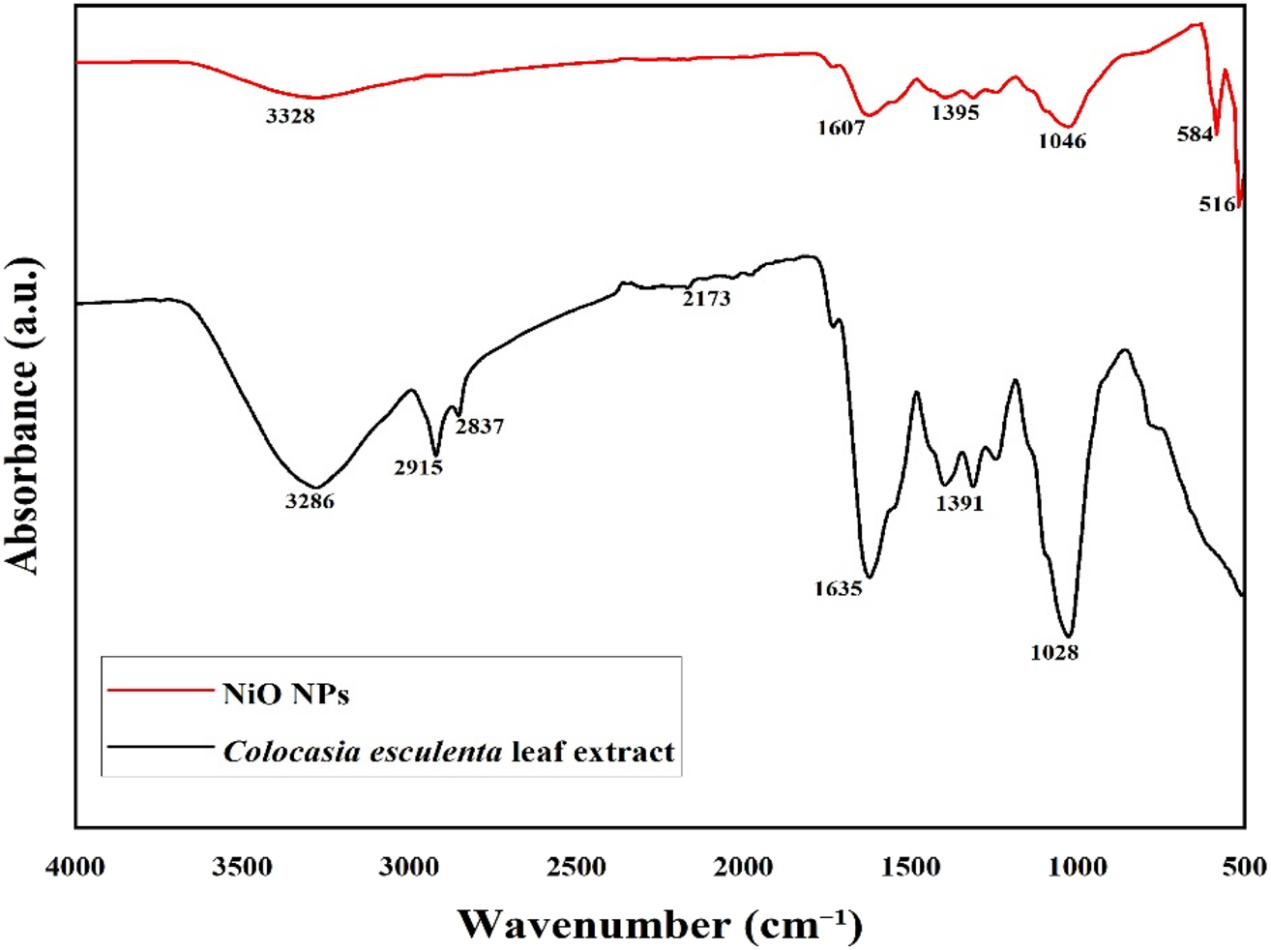
FT-IR spectra of the *Colocasia esculenta* leaf extract and green-synthesized NiO NPs.

The functional groups that were responsible for the reduction of NiO NPs were identified using FTIR spectroscopy. Fig. 4 displays the FTIR spectra of *Colocasia esculenta* leaf extract and the green-synthesized NiO NPs recorded in the 400–4000 cm^−1^ range.

The leaf extract exhibited peaks at 3286, 2915, 2837, 2173, 1635, 1391, and 1028 cm^−1^ for the O–H stretching of phenols and alcohols, C–H stretching of alkanes, N–C=O stretching of amides, C–H bending vibrations, and C–N stretching of amines. These functional groups are engaged in reducing Ni^2+^ ions and stabilizing the nanoparticles.

The FTIR spectrum of NiO NPs showed bands at 3328, 1607, 1395, 1046, 584, and 516 cm^−1^. The peaks at 3328 and 1607 cm^−1^ correspond to the O–H and C=O vibrations of adsorbed phytochemicals, while the features at 1395 and 1046 cm^−1^ represent the C–H and C–O vibrations of the residual organic moieties, respectively. The peaks at 584 and 516 cm^−1^ are attributed to the Ni–O stretching and bending vibrations, respectively, indicating the successful formation of NiO NPs.^44,45^ Peaks in the NiO NP sample showed a modest shift and decrease in intensity.^46^ These results confirm that phytochemicals in the *Colocasia esculenta* leaf extract act as reducing agents, promoting the environmentally friendly synthesis of NiO nanoparticles.^47^

### 3.4 XRD analysis of NiO nanoparticles

**Fig. 5:**
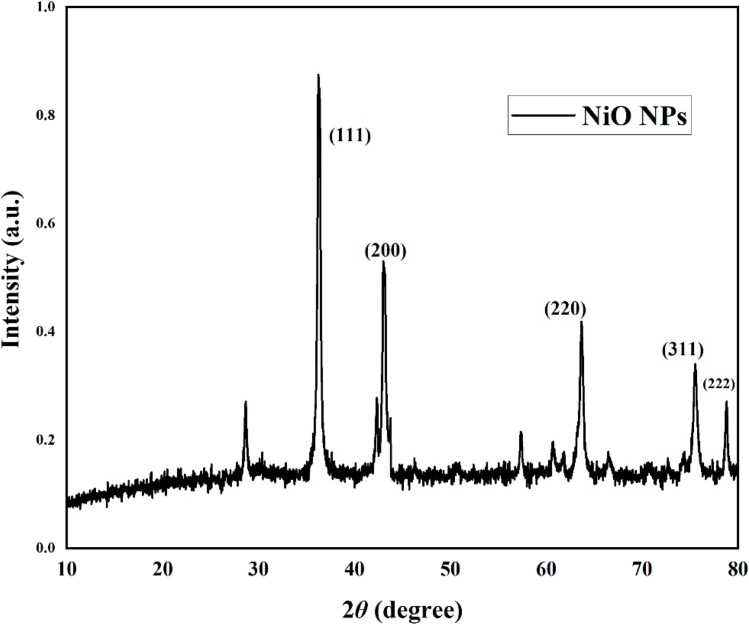
XRD pattern of the synthesized NiO nanoparticles.

The crystalline phase purity and structure of NiO NPs synthesized using the *Colocasia esculenta* leaf extract were investigated using X-ray diffraction in the 2*θ* range of 10–80° (Fig. 5). The detected diffraction peaks at 36.22°, 43.2°, 63.66°, 75.62°, and 78.84° correspond to the (111), (200), (220), (311), and (222) planes of FCC NiO NPs, respectively, in agreement with JCPDS card no. 47-1049.^18,48^ No additional peaks were detected, indicating the high phase purity. The sharpness of the peaks verifies that the nanoparticles were crystalline in nature, consistent with previously reported NiO NPs.^49^ Using the Debye–Scherrer equation (eqn (1)), it has been determined that the typical crystallite size of NiO NPs is 26.89 nm, falling within the reported literature range of 20–40 nm.^50,51^ Compared to previous reports using *Senna auriculata* extract, the synthesized NiO nanoparticles in this work exhibited slightly sharper and more intense peaks, suggesting improved crystallinity. The average crystallite size was smaller than the reported value (∼53 nm), indicating that the current synthesis method yields finer nanoparticles.^43^

### 3.5 SEM analysis of NiO nanoparticles

**Fig. 6:**
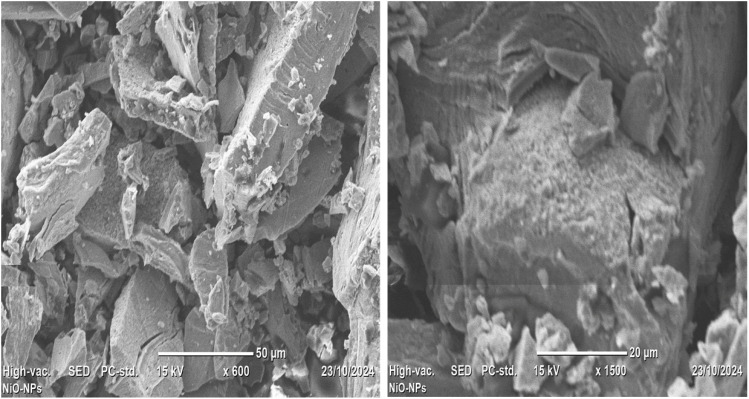
SEM images of the synthesized NiO nanoparticles at different magnifications.

Utilizing scanning electron microscopy (SEM), the surface morphology of the synthesized NiO nanoparticles was examined at magnifications of 600×, 1500×, and 4400× (Fig. 6). According to the images, the nanoparticles are primarily aggregated, most likely as a result of the drying/calcination process and high surface energy. The majority of the particles have inconsistent plate-like forms, which is in line with NiO nanostructures that have been previously observed.^51,52^

**Fig. 7:**
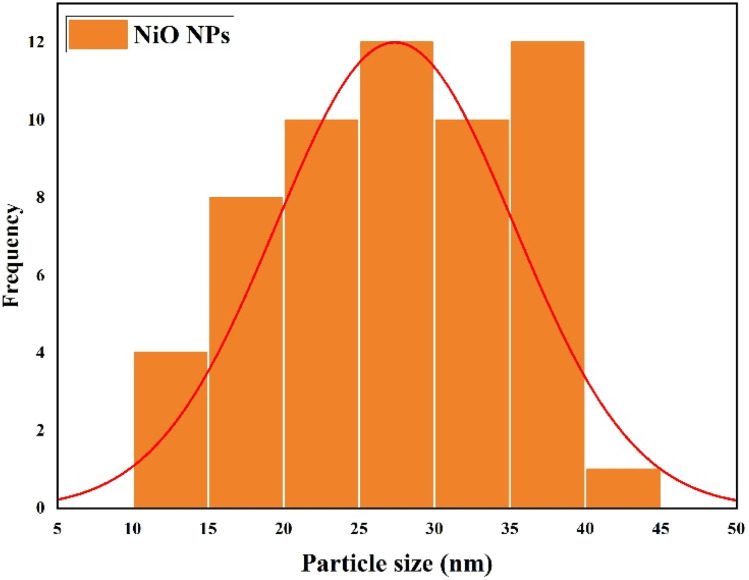
Particle size distribution of the synthesized NiO NPs obtained from SEM analysis (Fig. 6). The histogram shows the distribution of particle sizes measured using the ImageJ software (*N* = 57), ranging from 12 to 43 nm. The average particle size was 27.92 ± 7.97 nm.

A polycrystalline structure was seen, with some particles aggregating together as a result of smaller units overlapping or clustering. The particle sizes, measured using ImageJ software, ranged from 12 to 43 nm (Fig. 7), with an average size of approximately 27.92 ± 7.97 nm (*N* = 57). This is consistent with the size of the crystallites, as determined by XRD examination. This size is smaller than that reported by Ganesan *et al.*, where bio-fabricated NiO NPs ranged from 43 to 47 nm. The smaller particle size in the present study may enhance the surface area, contributing to the improved photocatalytic and antibacterial performance observed.^53^ It is anticipated that the NiO NPs nanoscale size and aggregated shape will improve the surface reactivity, which is advantageous for photocatalytic and antibacterial applications.

### 3.6 Zeta potential measurements

**Fig. 8:**
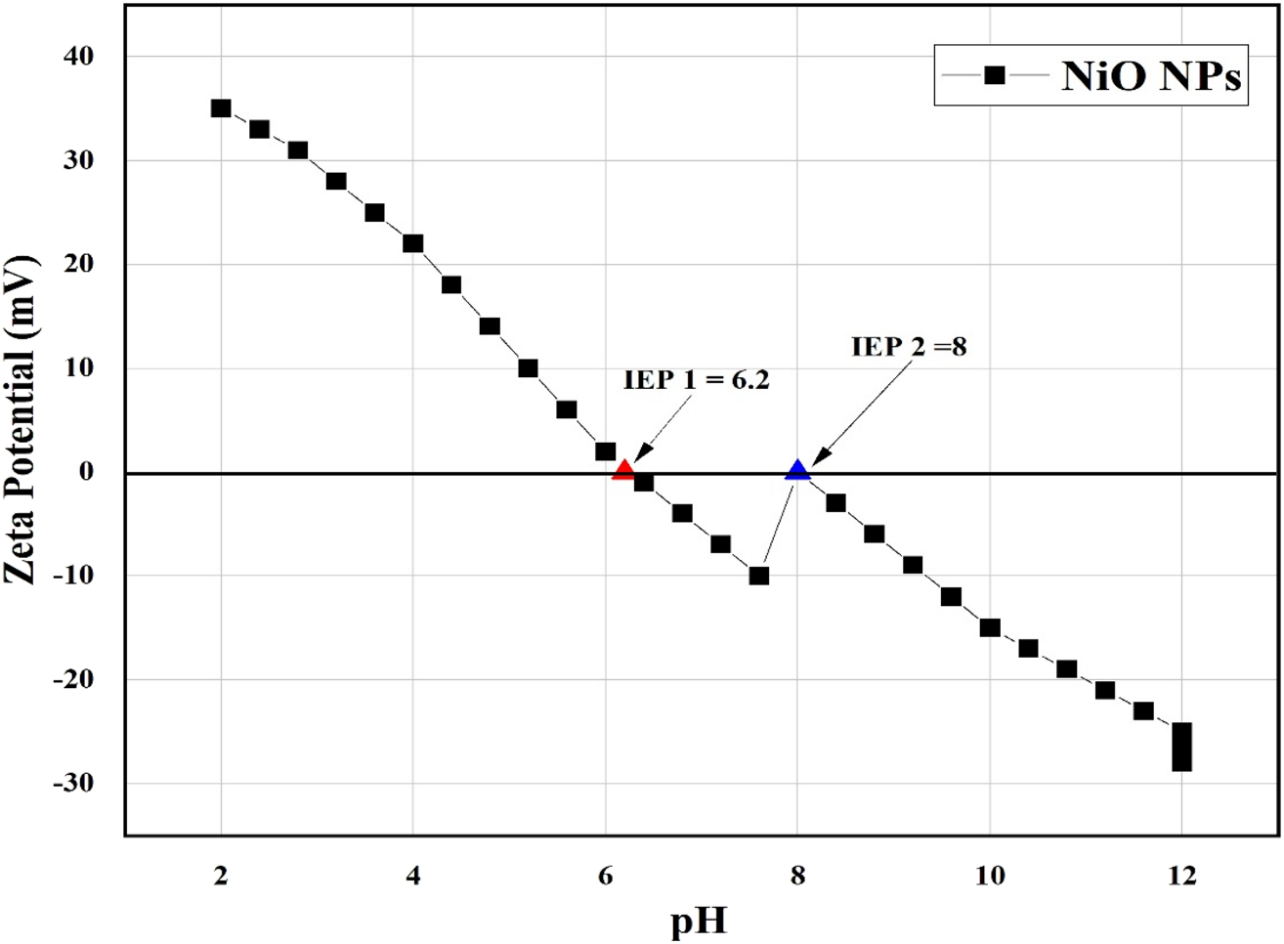
Zeta potential of NiO nanoparticles as a function of pH, showing two isoelectric points at pH 6.2 and 8.

Zeta potential studies were conducted as a function of pH to determine the surface charge of the synthesized NiO NPs. The results show two isoelectric points (IEPs) at pH 6.2 and 8, which may arise from different surface sites, such as hydroxyl groups, or residual ions from the synthesis process. The nanoparticles exhibit positive zeta potential values below the IEPs and negative values above, indicating that the colloidal stability is highest at pH values far from the IEPs. These findings are important for understanding the adsorption behavior of methylene blue and the photocatalytic performance of the nanoparticles^54^ (Fig. 8).

### 3.7 Antimicrobial activity

**Fig. 9:**
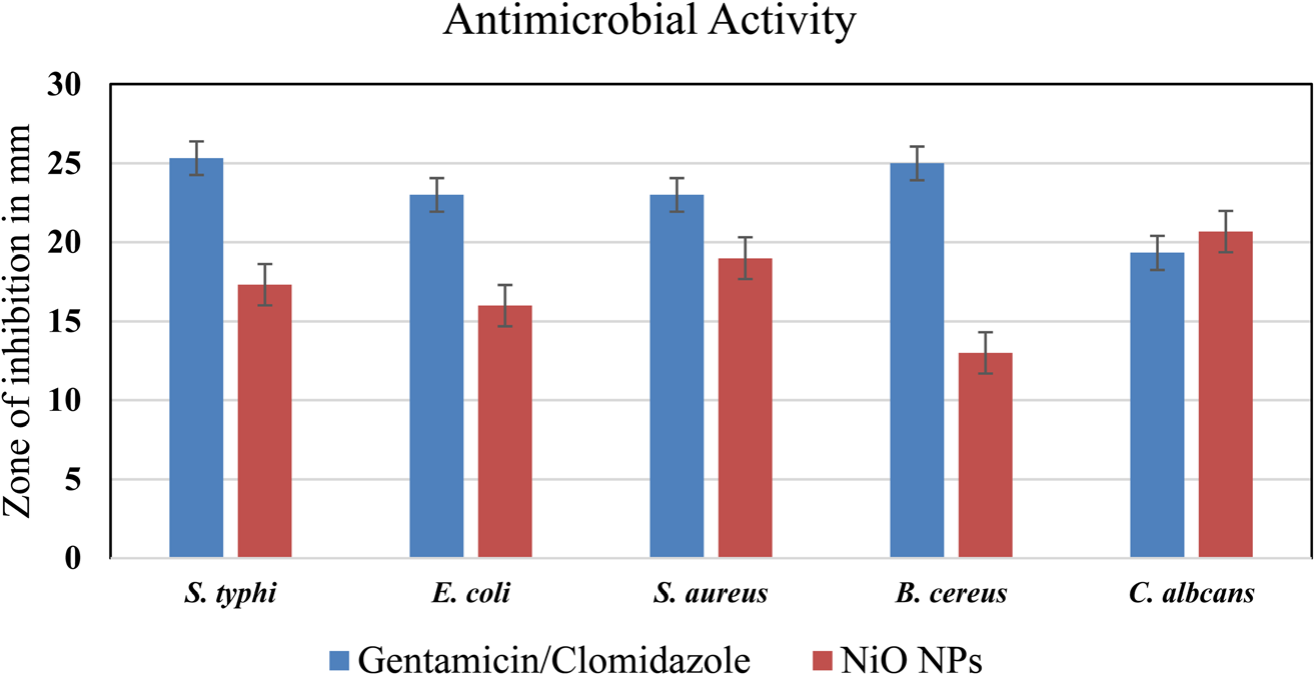
Antimicrobial activity of NiO NPs against bacterial and fungal strains.

Using the agar well diffusion method, the antibacterial and antifungal properties of green-synthesized NiO NPs were assessed against the fungal strain *C. albicans*, Gram-positive bacteria (*B. cereus*, *S. aureus*), and Gram-negative bacteria (*S. typhi*, *E. coli*). Gentamicin and clotrimazole were used as positive controls and DMSO was employed as a negative control. NiO NPs demonstrated significant antimicrobial activity, with the zone of inhibition (ZOI) increasing with the nanoparticle concentration (25–75 mg mL^−1^). NiO NPs typically show antibacterial activity at lower concentrations (low mg mL^−1^). The effect observed here at 75 mg mL^−1^ likely reflects the assay type, nanoparticle aggregation, and strain susceptibility.^55^ At 75 mg mL^−1^, the nanoparticles exhibited the highest antibacterial effect against *S. aureus* (19.00 ± 0.94 mm) and *S. typhi* (17.33 ± 0.62 mm), moderate activity against *E. coli* (16.00 ± 0.82 mm) and *B. cereus* (13.00 ± 58 mm), and strong antifungal activity against *C. albicans* (20.67 ± 0.58 mm) (Table 3 and Fig. 9).

**Table 3:** Zone of inhibition of the synthesized NiO nanoparticles (mean ± SD, *n* = 3)

Strain	Gentamicin/Clomidazole (+ve control)	DMSO (−ve control)	NiO NPs
*S. typhi*	25.33 ± 0.58	—	17.33 ± 0.62
*E. coli*	23.00 ± 1.00	—	16.00 ± 0.82
*S. aureus*	23.00 ± 0.58	—	19.00 ± 0.94
*B. cereus*	25.00 ± 1.00	—	13.00 ± 0.58
*C. albicans*	19.33 ± 0.58		20.67 ± 0.58

The structural variations of the bacterial cell walls are responsible for the reported variances in antibiotic activity. The outer lipopolysaccharide layer of Gram-negative bacteria, such as *E. coli* and *S. typhi*, prevents nanoparticle penetration. However, Gram-positive bacteria (*S. aureus* and *B. cereus*) have a thicker peptidoglycan covering with many pores, which makes it easier for nanoparticles to enter and disrupt the cell membranes, ultimately leading to bacterial death.^56^ The production of the reactive oxygen species (ROS) and small particle size enables better membrane penetration, which results in microbial cell damage and oxidative stress, amplifying the antibacterial action of NiO NPs.^57,58^

### 3.8 Photocatalytic activity

Under normal sun irradiation, methylene blue (MB) was used as a model pollutant to assess the photocatalytic activity of NiO NPs. Degradation of MB was monitored by UV-vis spectroscopy at 665 nm.^28,59^ A noticeable color shift from blue to almost colorless and a slow decrease in absorption intensity over time (10–80 min) suggested effective degradation. The presence of NiO NPs resulted in 96% MB degradation after 80 minutes of exposure to visible light, whereas the absence of nanoparticles caused very little degradation. This performance is slightly greater than that reported by Sayoud *et al.* for NiO NPs, which achieved 93%.^54^ This study achieved 96% degradation within 80 minutes, which is comparable to the 97% degradation reported by Al-Zaqri *et al.* using NiO nanoparticles biosynthesized with *Senna auriculata* flower extract in 90 minutes. The slightly faster degradation observed in the present study may be attributed to the smaller particle size and higher surface area, which enhance the generation of reactive species and improve catalytic efficiency.^43^ Effective separation of electrons and holes on the NiO NP surfaces and plasmonic stimulation in the presence of visible light result in a strong affinity for the dye molecule for adsorption, causing strong phytochemical activity.

### 3.9 Optimization of the photocatalytic parameters

#### 3.9.1 Dosage of the catalyst

**Fig. 10:**
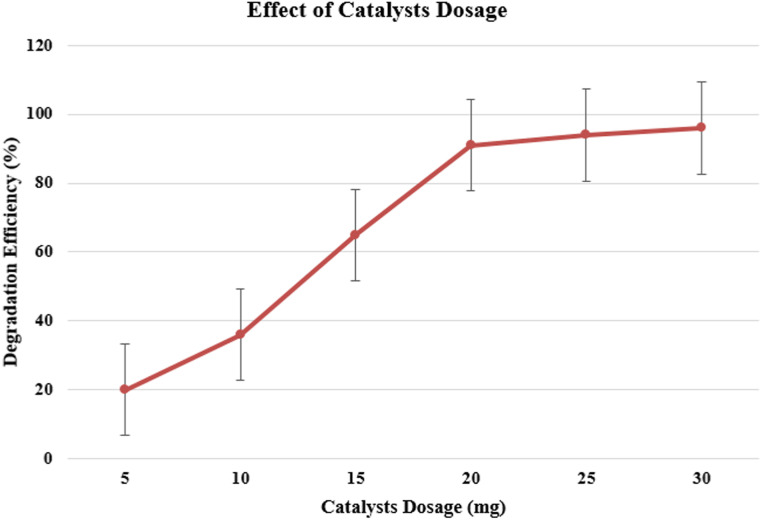
Effect of catalyst dosage on the degradation efficiency of methylene blue (MB).

Because there were more active sites and hydroxyl radicals formed, degradation improved from 20% to 96% when NiO NPs were increased from 5 mg to 30 mg (Fig. 10), respectively. Light scattering caused doses beyond 30 mg to lose efficiency.

#### 3.9.2 Point of zero charge (PZC)

**Fig. 11:**
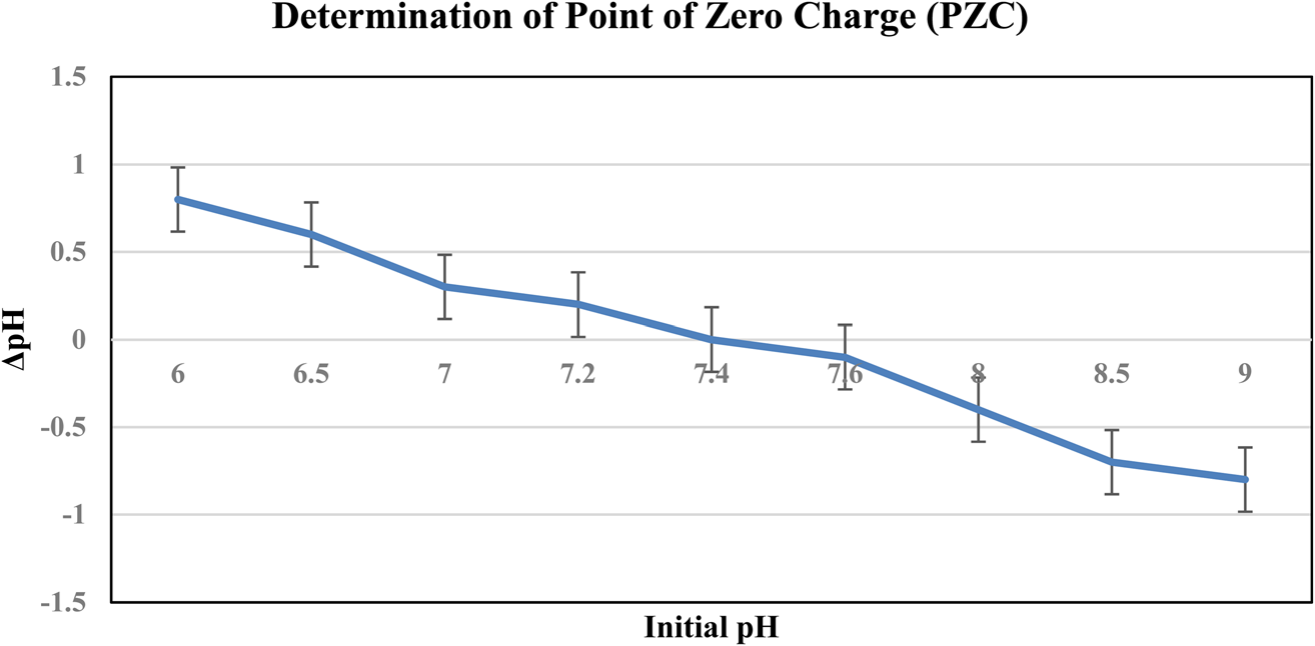
ΔpH as a function of the initial pH for determining the point of zero charge (PZC) of the catalyst (PZC ≈ 7.4).

The NiO nanoparticles show two IEPs at pH 6.2 and 8 due to different surface sites. The overall PZC is 7.4, which differs from the individual IEPs because the PZC represents the net surface neutrality, whereas the IEP reflects the charge at the slipping plane. The catalyst surface was negatively charged at pH > PZC, which improved the photocatalytic activity and facilitated the adsorption of cationic MB (Fig. 11).

#### 3.9.3 Effect of pH

**Fig. 12:**
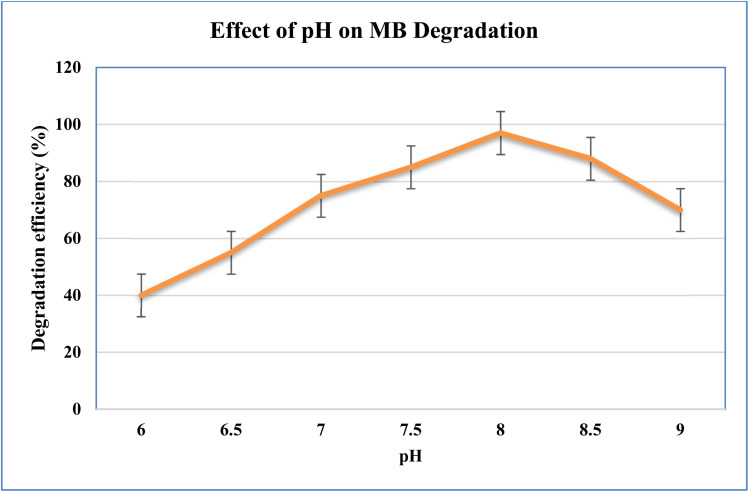
Effect of pH on the photocatalytic degradation of MB.

At pH 8, the maximum MB degradation (96%) was attained. Electrostatic repulsion decreased the adsorption at lower pH levels, whereas metal hydroxide production at higher pH values (>8) decreased the efficiency (Fig. 12).

#### 3.9.4 Initial dye concentration

**Fig. 13:**
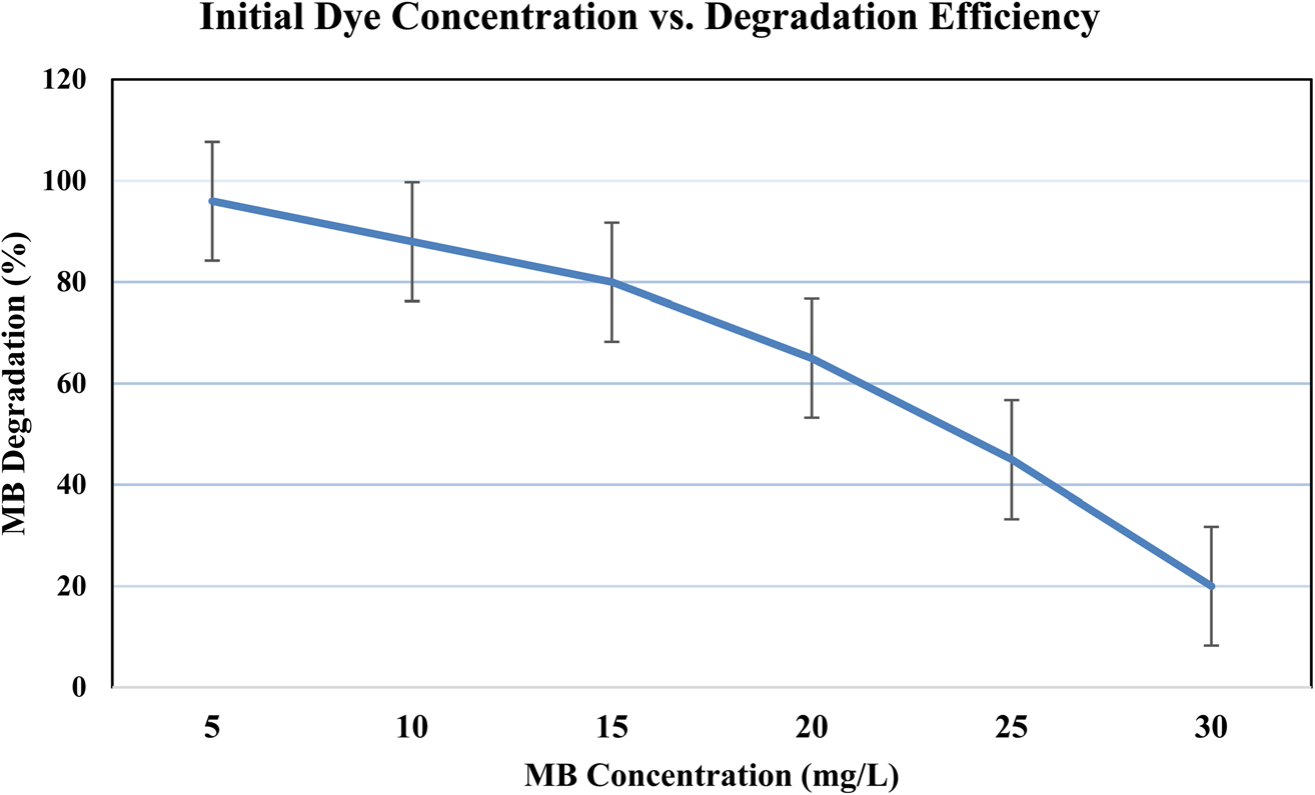
Effect of initial dye concentration on the photocatalytic degradation of methylene blue (MB).

While greater doses (30 mg L^−1^) decreased the degradation to 20% because of light absorption by the excess dye, low concentrations of MB (5 mg L^−1^) allowed for efficient photon penetration, resulting in 96% destruction after 80 minutes (Fig. 13).

#### 3.9.5 Contact time

**Fig. 14:**
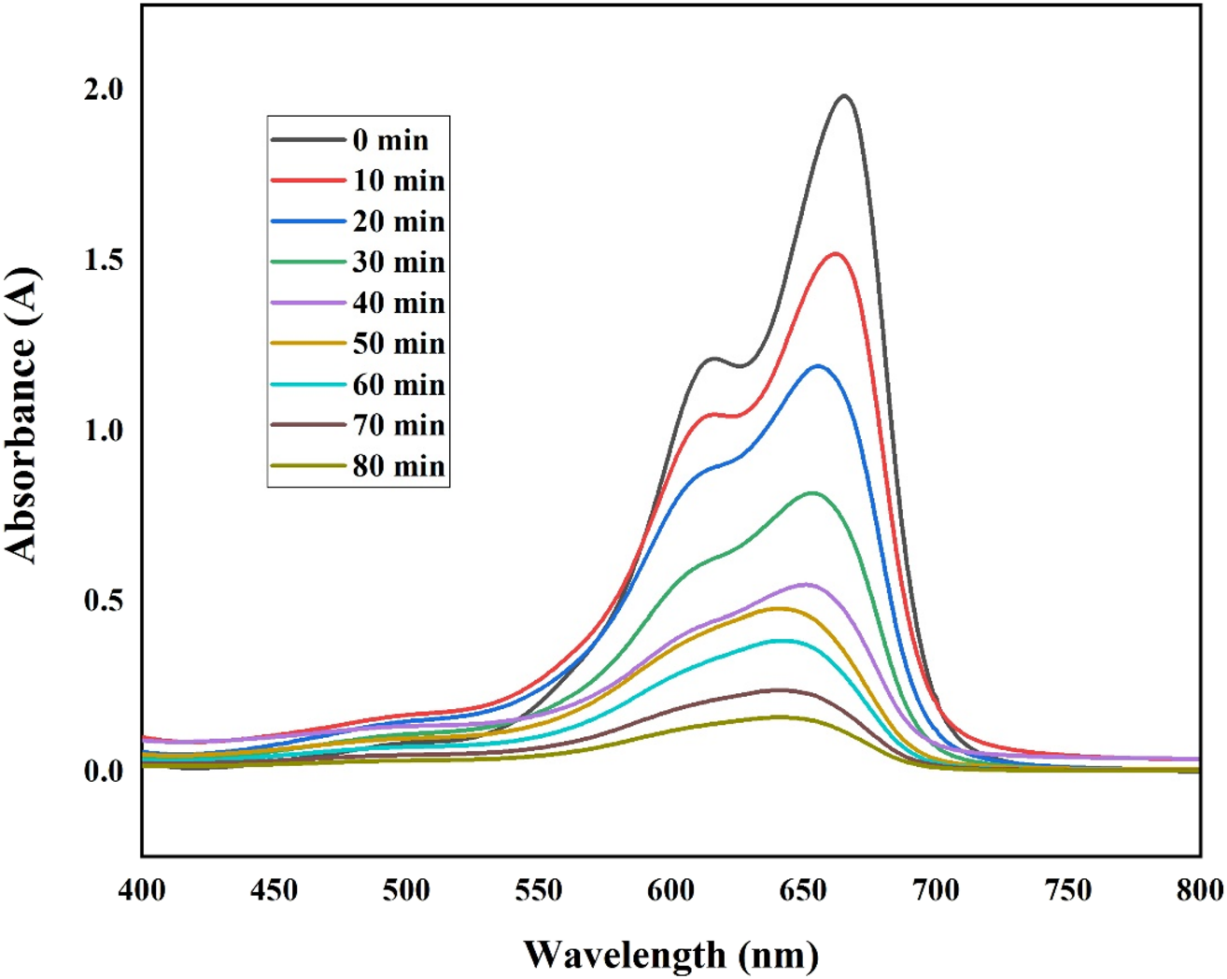
UV-vis spectra of MB degradation over time using NiO NPs.

According to the UV-vis spectra (Fig. 14), the MB absorbance rapidly dropped over the course of 80 minutes, reaching ∼92% degradation, demonstrating NiO NPs' effective photocatalytic activity.

#### 3.9.6 Reusability

**Fig. 15:**
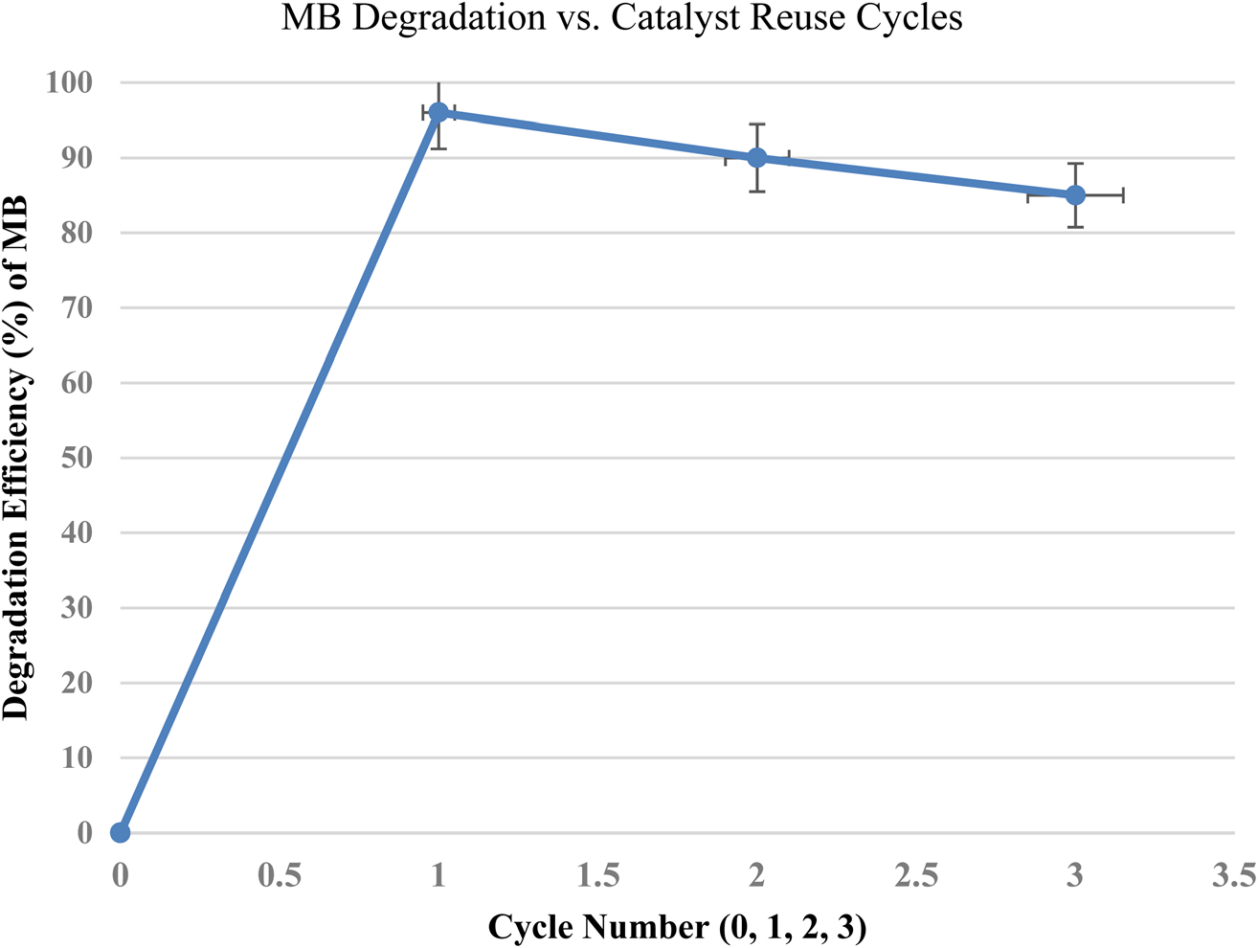
Photocatalytic degradation efficiency of methylene blue (MB) over three consecutive cycles using NiO NPs.

The catalyst was subjected to three reuse cycles under the same conditions (15 mg, 50 mL MB, pH 8, 80 min). The reported algae-mediated NiO nanoparticles showed good catalytic stability over three consecutive reuse cycles.^54^ Similarly, NiO NPs produced in the present study exhibited excellent reusability, maintaining photocatalytic efficiencies from 96% in the first cycle to approximately 85% after the third cycle. This confirms the structural stability and photocorrosion resistance of the catalyst during repeated use, indicating its potential for practical wastewater treatment applications (Fig. 15).

## 4 Conclusion

In summary, *Colocasia esculenta* leaf extract was effectively used to synthesize nickel oxide (NiO) nanoparticles, providing a productive, sustainable, and environmentally friendly green synthesis method. Crystalline NiO nanoparticles with nanoscale dimensions were formed, as validated by structural and morphological characterization using XRD and SEM. The produced nanoparticles exhibited notable antibacterial activity against multiple microorganisms, including *S. typhi* (17.33 ± 0.62 mm), *E. coli* (16.00 ± 0.82 mm), *S. aureus* (19.00 ± 0.94 mm), *B. cereus* (13.00 ± 0.58 mm), and *C. albicans* (20.67 ± 0.58 mm). In addition, the nanoparticles demonstrated excellent photocatalytic activity, achieving 96% methylene blue degradation under visible light. After three cycles, 85% catalytic activity was maintained, indicating the stability, reusability, and potential of NiO NPs as an environmentally benign photocatalyst. These findings demonstrate the dual functionality of the biosynthesized NiO NPs, highlighting their potential for environmental remediation and antimicrobial applications. Overall, this study shows the dual functioning of NiO nanoparticles produced using the leaf extract from *Colocasia esculenta*, indicating their suitability for use in environmental and medicinal domains.

## Conflicts of interest

The authors declare no conflict of interest.

## Supplementary Material

RA-016-D5RA08840B-s001

## Data Availability

All data generated or analyzed during this study are included in the published article and its supplementary information (SI) files. Additional datasets generated and/or analyzed during the current study are available from the corresponding author upon reasonable request. Supplementary information is available. See DOI: https://doi.org/10.1039/d5ra08840b.

## References

[cit1] Zeid E. F. A., Ibrahem I. A., Mohamed W. A. A., Ali A. M. (2020). Study the influence of silver and cobalt on the photocatalytic activity of copper oxide nanoparticles for the degradation of methyl orange and real wastewater dyes. Mater. Res. Express.

[cit2] Taha A., Gouda S. A. (2025). Eco-friendly dye removal: Impact of dyes on aquatic and human health and sustainable fungal treatment approaches, Egypt. J. Aquat. Biol. Fish..

[cit3] Liu Q. (2020). Pollution and treatment of dye waste-water. IOP Conf. Ser. Earth Environ. Sci..

[cit4] Sarkar P. A. K. , Tortora G. and Johnson I., Photodegradation, Fairchild Books Dict. Text., 2022, 10.5040/9781501365072.12105

[cit5] Kato S., Kansha Y. (2024). Comprehensive review of industrial wastewater treatment techniques. Environ. Sci. Pollut. Res..

[cit6] Firisa S. G., Muleta G. G., Yimer A. A. (2022). Synthesis of nickel oxide nanoparticles and copper-doped nickel oxide nanocomposites using Phytolacca dodecandra L'Herit leaf extract and evaluation of its antioxidant and photocatalytic activities. ACS Omega.

[cit7] Krishna P. G., Chandra Mishra P., Naika M. M., Gadewar M., Ananthaswamy P. P., Rao S., Boselin Prabhu S. R., Yatish K. V., Nagendra H. G., Moustafa M., Al-Shehri M., Jha S. K., Lal B., Stephen Santhakumari S. M. (2022). Photocatalytic activity induced by metal nanoparticles synthesized by sustainable approaches: a comprehensive review. Front. Chem..

[cit8] Truong V. K., Truong N. P., Rice S. A. (2021). Antibacterial activity of nanoparticles. Nanomaterials.

[cit9] Ding D., Wang B., Zhang X., Zhang J., Zhang H., Liu X., Gao Z., Yu Z. (2023). The spread of antibiotic resistance to humans and potential protection strategies. Ecotoxicol. Environ. Saf..

[cit10] Ghirardello M., Ramos-Soriano J., Galan M. C. (2021). Carbon dots as an emergent class of antimicrobial agents. Nanomaterials.

[cit11] Khan M. I., Nawaz M., Tahir M. B., Iqbal T., Pervaiz M., Rafique M., Aziz F., Younas U., Alrobei H. (2020). Inorg. Chem. Commun..

[cit12] Qadri H., Shah A. H., Mir M. (2020). Novel strategies to combat the emerging drug resistance in human pathogenic microbes. Curr. Drug Targets.

[cit13] Ijaz I., Gilani E., Nazir A., Bukhari A. (2020). Detail review on chemical, physical and green synthesis, classification, characterizations and applications of nanoparticles. Green Chem. Lett. Rev..

[cit14] Shabani L., Abbasi M., Azarnew Z., Amani A. M., Vaez A. (2023). Neuro-nanotechnology: diagnostic and therapeutic nano-based strategies in applied neuroscience. Biomed. Eng. Online.

[cit15] Singh V. , Yadav P. and Mishra V., Recent advances on classification, properties, synthesis, and characterization of nanomaterials, in Green Synthesis of Nanomaterials for Bioenergy Applications, 2020, pp. 83–97, 10.1002/9781119576785.ch3

[cit16] Shaikhaldein H. O., Al-qurainy F., Khan S., Nadeem M., Tarroum M., Salih A. M., Gaafar A. R. Z., Alshameri A., Alansi S., Alenezi N. A., Alfarraj N. S. (2021). Biosynthesis and characterization of ZnO nanoparticles using Ochradenus arabicus and their effect on growth and antioxidant systems of Maerua oblongifolia. Plants.

[cit17] Gebremichael T. E., Muleta G. G., Tadele K. T. (2025). Green synthesis of Ag-doped CuO nanocomposites using honey solution for evaluation of their antimicrobial and antioxidant activities. Curr. Nanomater..

[cit18] Bulla M., Kumar V., Devi R., Kumar S., Sisodiya A. K., Dahiya R., Mishra A. K. (2024). Natural resource-derived NiO nanoparticles via Aloe vera for high-performance symmetric supercapacitor. Sci. Rep..

[cit19] Ezhilarasi A., Vijaya J., Kaviyarasu K., John Kennedy L., Ramalingam R. J., Al-Lohedan H. A. (2018). Green synthesis of NiO nanoparticles using Aegle marmelos leaf extract for the evaluation of in-vitro cytotoxicity, antibacterial and photocatalytic properties. J. Photochem. Photobiol. B: Biol..

[cit20] Robi Z. H., Nemera D. J., Gebremichael T. E., Muleta G. G. (2025). Dual-functional nitrogen-doped carbon dot/copper oxide nanocomposites for electrochemical sensing of ascorbic acid and antimicrobial applications. BMC Chem.

[cit21] Bilici Z., Koc E. O., Özdemir S., Yalçın M. S., Filiz V., Dizge N. (2025). Green synthesis of nickel oxide nanoflowers from cherry straw extract and preparation of antibacterial PES ultrafiltration membrane. Int. J. Environ. Sci. Technol..

[cit22] Sanni S. E. , Oni B. A., Okoro E. E. and Pandya S., Recent Advances in the Use of Biogenic Nanomaterials and Photocatalysts for Wastewater Treatment: Challenges and Future Prospects, 2024, 6, 10.3389/fnano.2024.1469309

[cit23] Zubair M. W., Imran A., Islam F., Afzaal M., Saeed F., Zahra S. M., Akhtar M. N., Noman M., Ateeq H., Aslam M. A., Mehta S., Shah M. A., Awuchi C. G. (2023). Functional profile and encapsulating properties of Colocasia esculenta (Taro). Food Sci. Nutr..

[cit24] Shakor S. R. , Fardood S. T. and Naghipour A., Green Synthesis and Characterization of NiO Nanoparticles with Enhanced Sonocatalytic Activity, 2025, 15, 1–10

[cit25] Kannan K., Radhika D., Sadasivuni K. K., Reddy K. R., Raghu A. V. (2020). Nanostructured metal oxides and its hybrids for photocatalytic and biomedical applications. Adv. Colloid Interface Sci..

[cit26] Kumari S. C., Dhand V., Padma P. N. (2021). Green synthesis of metallic nanoparticles: a review. Nanomaterials.

[cit27] Alvarez-Suarez J. M., Tulipani S., Romandini S., Bertoli E., Battino M. (2010). Contribution of honey in nutrition and human health: a review. Mediterr. J. Nutr. Metab.

[cit28] Fagier M. A. (2021). Plant-mediated biosynthesis and photocatalysis activities of zinc oxide nanoparticles: a prospect towards dyes mineralization. J. Nanotechnol.

[cit29] Hong S.-J., Mun H.-J., Kim B.-J., Kim Y.-S. (2021). Characterization of nickel oxide nanoparticles synthesized under low temperature. Micromachines.

[cit30] Eshtu A., Endris Y., Lakew S. (2024). Biogenic mediated green synthesis of NiO nanoparticles for adsorptive removal of lead from aqueous solution. Heliyon.

[cit31] Khan M. A., Nayan N., Shadiullah, Ahmad M. K., Soon C. F. (2020). Surface study of CuO nanopetals by advanced nanocharacterization techniques with enhanced optical and catalytic properties. Nanomaterials.

[cit32] Vijayaraghavan K., Ashokkumar T. (2017). Plant-mediated biosynthesis of metallic nanoparticles: a review of literature, factors affecting synthesis,
characterization techniques and applications. J. Environ. Chem. Eng..

[cit33] Roshan Balasooriya E. , Jayasinghe C. D., Jayawardena U. A., Weerakkodige R., Ruwanthika D., Mendis De Silva R. and Udagama P. V., New Era Of Safe Nanotechnology, 2017

[cit34] Vasilev K. (2023). Antibacterial applications of nanomaterials. Nanomaterials.

[cit35] Sathiyavimal S., Vasantharaj S., Bharathi D., Saravanan M., Manikandan E., Kumar S. S., Pugazhendhi A. (2018). Biogenesis of copper oxide nanoparticles (CuONPs) using Sida acuta and their incorporation over cotton fabrics to prevent the pathogenicity of Gram negative and Gram positive bacteria. J. Photochem. Photobiol. B: Biol..

[cit36] Preethi D. R. A., Philominal A. (2022). Green synthesis of pure and silver doped copper oxide nanoparticles using Moringa oleifera leaf extract. Mater. Lett. X.

[cit37] Jayachandran A., Aswathy T. R., Nair A. S. (2021). Green synthesis and characterization of zinc oxide nanoparticles using Cayratia pedata leaf extract. Biochem. Biophys. Rep..

[cit38] Burange P. J., Tawar M. G., Bairagi R. A., Malviya V. R., Sahu V. K., Shewatkar S. N., Sawarkar R. A., Mamurkar R. R. (2021). Synthesis of silver nanoparticles by using Aloe vera and Thuja orientalis leaves extract and their biological activity: a comprehensive review. Bull. Natl. Res. Cent..

[cit39] Pelegrino M. T. , Kohatsu M. Y., Seabra A. B., Rolim R., De Jesus T. A., Batista B. L. and Lange C. N., Effects of copper oxide nanoparticles on growth of lettuce (Lactuca sativa L.) seedlings and possible implications of nitric oxide in their antioxidative defense, 2020 10.1007/s10661-020-8188-3 32166379

[cit40] Haider A. J., Al-Anbari R., Sami H. M., Haider M. J. (2019). Photocatalytic activity of nickel oxide. J. Mater. Res. Technol..

[cit41] Suresh L., Snega R., Geetha Sravanthy P., Saravanan M. (2024). Phytosynthesis of nickel oxide nanoparticles and their antioxidant and antibacterial efficacy studies. Cureus.

[cit42] Ravishankar T. N., Ananda A., Shilpa B. M., Adarsh J. R. (2025). Eco-friendly synthesis of NiO and Ag/NiO nanoparticles: applications in photocatalytic and antibacterial activities. R. Soc. Open Sci..

[cit43] Al-Zaqri N., Umamakeshvari K., Mohana V., Muthuvel A. (2022). Green synthesis of nickel oxide nanoparticles and its photocatalytic degradation and antibacterial activity. J. Mater. Sci.: Mater. Electron..

[cit44] Khairnar S. D., Shrivastava V. S. (2019). Facile synthesis of nickel oxide nanoparticles for the degradation of methylene blue and rhodamine B dye: a comparative study. J. Taibah Univ. Sci..

[cit45] Khule R. N. and Arts S. S., Study The Structural, Functional Groups And Morphological Properties Of Co-Precipitated Technique Synthesised Nio Nanoparticles, 2022, 7, 740–742

[cit46] Hong S.-J., Mun H.-J., Kim B.-J., Kim Y.-S. (2021). Characterization of nickel oxide nanoparticles synthesized under low temperature. Micromachines.

[cit47] Thatyana M., Dube N. P., Kemboi D., Manicum A. L. E., Mokgalaka-Fleischmann N. S., Tembu J. V. (2023). Advances in phytonanotechnology: a plant-mediated green synthesis of metal nanoparticles using Phyllanthus plant extracts and their antimicrobial and anticancer applications. Nanomaterials.

[cit48] Zemieche A., Chetibi L., Hamana D., Achour S., Noto V. D. (2024). Symmetric and asymmetric supercapacitor fabrication based on green synthesized NiO nanoparticles and graphene. Colloid J..

[cit49] Goumri-Said S., Turgut G., Kanoun M. B. (2023). Lu doping nickel oxide thin films using sol-gel spin coated and density functional theory: optoelectronic and magnetic properties. Heliyon.

[cit50] Uddin S., Bin Safdar L., Anwar S., Iqbal J., Laila S., Abbasi B. A., Saif M. S., Ali M., Rehman A., Basit A., Wang Y., Quraishi U. M. (2021). Green synthesis of nickel oxide nanoparticles from Berberis balochistanica stem for investigating bioactivities. Molecules.

[cit51] Srihasam S., Thyagarajan K., Korivi M., Lebaka V. R., Mallem S. P. R. (2020). Phytogenic generation of NiO nanoparticles using Stevia leaf extract and evaluation of their in-vitro antioxidant and antimicrobial properties. Biomolecules.

[cit52] Ashraf M. A., Peng W., Zare Y., Rhee K. Y. (2018). Effects of size and aggregation/agglomeration of nanoparticles on the interfacial/interphase properties and tensile strength of polymer nanocomposites. Nanoscale Res. Lett..

[cit53] Monisha G., Rejo Jeice A., Rajaram P. (2024). Annealing effects on bio-fabricated nickel oxide nanoparticles for environmental remediation: Photocatalytic dye degradation and antimicrobial activity. J. King Saud Univ. Sci..

[cit54] Sayoud N., Bouchair A., Khen O., Laib S., Boudellioua H., Hadji F., Zoukel A., Touati H. (2025). Sustainable NiO nanoparticles photocatalysts for efficient methylene blue removal: synthesis, characterization, and kinetic studies. React. Kinet. Mech. Catal..

[cit55] Sukumaran J., Priya M., Venkatesan R., Sathiasivan K., Khan M. R., Kim S.-C. (2025). Green synthesis of nickel oxide nanoparticles using leaf extract of Aegle marmelos and their antibacterial, antioxidant and in vitro cytotoxicity activity. Microsc. Res. Tech..

[cit56] Dye C. R. , Murugalakshmi M., Standard T. and Rajaratnam F., Nickel Oxide Nanoparticles Synthesis By Chemical Reduction Technique, 2022, 13, 4934–4941, 10.13040/IJPSR.0975-8232.13(12).4934-41

[cit57] Tamesgen T., Ameya M. A., Sisay G., Yuanqi L., Kai Z., Beyene T. T. (2025). Harnessing the power of S/N-doped NiO nanoparticles through bandgap tuning to achieve enhanced photocatalytic and antibacterial performances. Sci. Rep..

[cit58] Shnawa B. H., Jalil P. J., Hamad S. M., Ahmed M. H. (2022). Antioxidant, protoscolicidal, hemocompatibility, and antibacterial activity of nickel oxide nanoparticles synthesized by Ziziphus spina-christi. Bionanoscience.

[cit59] Liang Y., Bin Chan Y., Aminuzzaman M., Shahinuzzaman M., Djearamane S., Thiagarajah K., Leong S.-Y., Wong L.-S., Tey L.-H. (2025). Green synthesis and characterization of copper oxide nanoparticles from durian (Durio zibethinus) husk for environmental applications. Catalysts.

