# Supplementary material for: Bio-derived NiO nanoparticles from Colocasia esculenta leaf extract with enhanced antibacterial activity and efficient photocatalytic degradation of methylene blue
Source: RSC Adv. 2026 Jan 15;16(3):2030–43. doi: 10.1039/d5ra08840b (PMC12805534; doi:10.1039/d5ra08840b)
Supplement: RA-016-D5RA08840B-s001 [file RA-016-D5RA08840B-s001.pdf]

Supplementary Information (SI) for RSC Advances.
This journal is © The Royal Society of Chemistry 2026

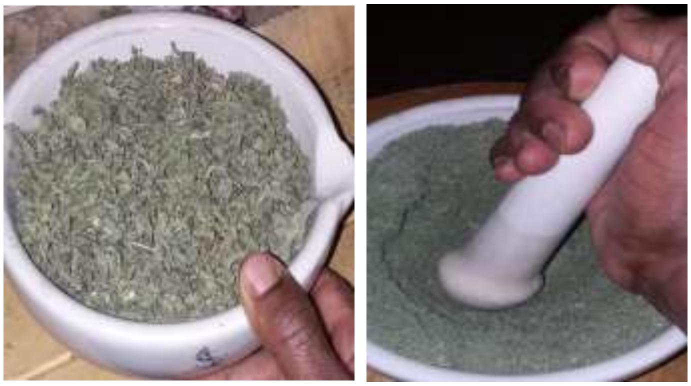

Figure S1. Representative photograph of the dried and powdered *Colocasia esculenta* leaves used as the starting material for extract preparation.

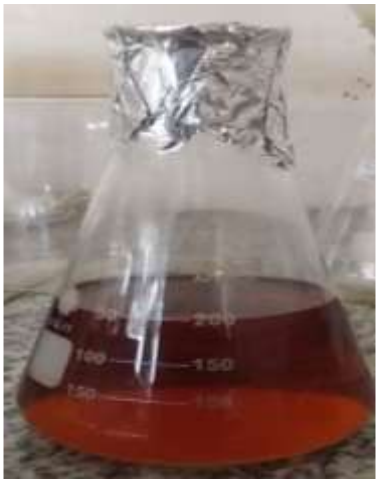

Figure S2. Photograph of the *Colocasia esculenta* leaf extract used for the synthesis of the NiO NPs.

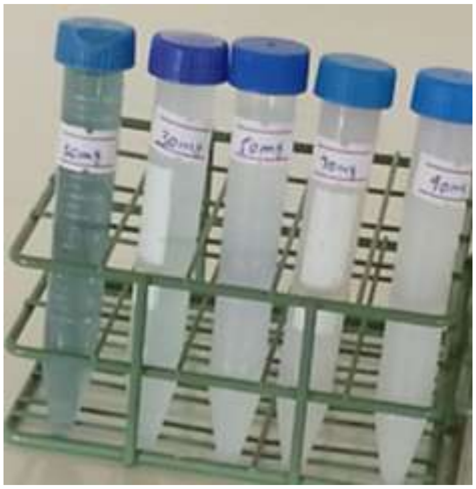

Figure S3. Time-dependent degradation of the target pollutant in the presence of the NiO NPs.
